# Efficacy of withdrawal or tapering of bDMARDs vs. standard regimen in axial spondyloarthritis patients: systematic review and meta-analysis informing the update of the Spanish Society of Rheumatology Guideline

**DOI:** 10.3389/fmed.2025.1621313

**Published:** 2025-09-10

**Authors:** Hye Sang Park, Petra Díaz del Campo, Maria Nieves Plana, Jessica Polo y La Borda, Mercedes Guerra-Rodríguez, Mireia Moreno, Juan D. Cañete

**Affiliations:** ^1^Rheumatology Department, Hospital de la Santa Creu i Sant Pau, Biomedical Research Institute (IIB Sant Pau), Universitat Autònoma de Barcelona, Barcelona, Spain; ^2^Research Unit, Spanish Society of Rheumatology (SER), Madrid, Spain; ^3^Health Technology Assessment Unit, Hospital Universitario Ramón y Cajal (IRYCIS), Madrid, Spain; ^4^Department of Surgery, Medical and Social Sciences, Faculty of Medicine and Health Sciences, University of Alcala, Madrid, Spain; ^5^CIBER Epidemiology and Public Health (CIBERESP), Madrid, Spain; ^6^Rheumatology Department, Hospital Central de la Defensa Gómez Ulla, Health Research Institute-Fundación Jiménez Díaz (IIS-FJD), Madrid, Spain; ^7^Department of Reumatology, Parc Taulí Hospital Universitari, Institut d'Investigació i Innovació Parc Taulí (I3PT-CERCA), Taulí de Sabadell, Spain; ^8^Department of Medicine, Universitat Autònoma de Barcelona, Sabadell, Spain; ^9^Arthritis Unit, Rheumatology Department, Hospital Clínic and IDIBAPS, Barcelona, Spain

**Keywords:** tapering, discontinuation, withdrawal, suspension, antirheumatic agents, disease modifying antirheumatic drugs, spondyloarthritis

## Abstract

**Objectives:**

To assess efficacy of withdrawal or tapering biologic disease-modifying antirheumatic drugs (bDMARDs) vs. maintaining a standard regimen in patients with axial spondyloarthritis (axSpA) to sustain remission.

**Methods:**

We conducted a systematic review of randomized controlled trials (RCTs) through July 2025, comparing treatment discontinuation or tapering against standard bDMARDs regimens in axSpA. We included RCTs of axSpA patients randomized to treatment interruption or tapering compared with standard treatment regimens. Outcomes measures included remission or flare measured by Ankylosing Spondylitis Disease Activity Score (ASDAS), Bath Ankylosing Spondylitis Disease Activity Index (BASDAI), and acute phase reactants. We rated the certainty of evidence using the Grading of Recommendations Assessment, Development and Evaluation (GRADE) system. We presented dichotomous outcomes as risk ratios (RR) with their 95% confidence intervals (CI). We used a random-effect model to perform a pooled analysis.

**Results:**

Eight RCTs involving 1,384 participants were analyzed. For those in sustained remission >6 months, withdrawal led to a significantly lower rate of inactive disease state (RR 0.58, CI 0.45–0.76; high certainty) and increased risks of flare (RR 1.79, CI 1.34–2.38; high certainty) and partial flares (RR 1.57, CI 1.25–1.97; high certainty) compared to the standard regimen. Patients with short-term remission <6 months and treatment withdrawal experienced significantly higher rates of flares (RR 0.41 CI 0.23–0.72, low certainty) and increased disease activity (RR 0.36 CI 0.15–0.86, low certainty). Tapering in 211 patients showed non-significant risk reductions in maintaining remission or low disease activity (RR 0.89, CI 0.66–1.18; moderate certainty).

**Conclusion:**

Treatment withdrawal reduces the likelihood of maintaining inactive or low disease activity. Tapering does not significantly compromise the maintenance of remission or low disease activity offering a safer alternative to complete treatment withdrawal.

## 1 Introduction

Over the past three decades, the primary goal of patient treatment in rheumatology has been tightly focused on disease control. However, in recent years, there has been a notable shift in interest toward achieving optimal control of disease comorbidities and enhancing patients' quality of life. While the American College of Rheumatology (ACR) guideline of 2019 does not recommend tapering or withdrawal of treatment (1), updated recommendations from the European League Against Rheumatism (EULAR) suggest that tapering strategies may be a viable approach (2). To date, systematic literature reviews (SLRs) examining the efficacy and safety of tapering and withdrawal strategies in axial spondyloarthritis (axSpA) have been published (3–5) including mostly studies prior to 2019, with a smaller pool of studies and patients, as well as increased heterogeneity in the findings.

To address these gaps and provide updated guidance, we have conducted a SLR and meta-analysis as part of the development process for updating the Spanish Society of Rheumatology's guideline for axial spondyloarthritis (ESPOGUIA) (6). We have incorporated more recent randomized controlled trials (RCTs) focusing on treatment withdrawal and tapering according to previous remission duration. We aim to provide clinicians with updated guidance informed by the latest evidence and tailored to the diverse needs of patients with axSpA.

The objective of this study was to evaluate the efficacy of withdrawal or tapering treatment compared to continuing a standard regimen of biologic disease-modifying antirheumatic drugs (bDMARDs) in maintaining remission in patients with axSpA. The results of this study are relevant to inform clinical decisions regarding long-term treatment of axAspA, which could lead to optimized patient outcomes and resource utilization in clinical practice.

## 2 Methods

Our results are reported, and our analyses conducted, in accordance with the guidelines of the Cochrane Collaboration and the Preferred Reporting Items for SLR and Meta-analysis (PRISMA) (7). This study, being a meta-analysis, involved the secondary use of existing, publicly available, and de-identified data. According to the European General Data Protection Regulation (GDPR), such studies do not require formal ethics approval. Therefore, our protocol was not reviewed by the Ethics Committee.

### 2.1 Inclusion criteria

The research question was formulated according to the Population, Intervention, Comparison, Outcome and Study design (PICOS) method, in which each of the items was defined as specified below.

We selected for inclusion randomized clinical trials including adult patients (≥18 years) diagnosed with axSpA.

Interventions included were: withdrawal (Placebo), dose reduction, or tapering (increased administration interval) of bDMARDs including Infliximab, Etanercept, Adalimumab, Certolizumab, Golimumab, Secukinumab, Ixekizumab, and Bimekizumab.

Comparison interventions included bDMARDs indicated for axSpA at their standard doses and administration intervals.

Outcomes of interest were: remission and low disease activity, defined by Ankylosing Spondylitis Disease Activity Score (ASDAS) with C-Reactive Protein (CRP); or Bath Ankylosing Spondylitis Disease Activity Index (BASDAI) <4 and low serum CRP concentration; disease flare according to ASDAS or BASDAI.

There were no restrictions on minimum follow-up time or sample size.

### 2.2 Search strategy

A thorough bibliographic search was conducted by a documentary specialist (MG) to identify studies meeting the inclusion criteria in the Pubmed (MEDLINE), EMBASE, and Cochrane Library databases up to July 2025.

Our search strategy included both medical subject headings (MeSH) and free text terms relevant to the population, intervention, comparison, and outcome framework. The MeSH terms used included: “axial spondyloarthritis” [Mesh] OR “spondylitis, Ankylosing” [Mesh] OR “axial SpA” AND “interrupt^*^” OR “discontin^*^” OR “termination” OR “withdraw^*^.”

Language restriction was not applied.

### 2.3 Study selection and data extraction

We included RCTs that evaluated the impact of withdrawal or tapering bDMARDs compared to the standard regimen on disease activity in patients with axSpA. Additionally, we included prior rigorous SLR to identify further studies relevant to our research question. Two reviewers (HSP and JPB) independently conducted an initial screening of titles and abstracts. Subsequently, the articles considered as potentially relevant were assessed in full-text. Any disagreements between the reviewers were resolved through consensus or consultation with expert reviewers (PDC or MNP). Data extracted included study design, population characteristics, interventions, comparators, sample size, and results. Additionally, authors were contacted via email to clarify uncertainties or to retrieve missing data.

### 2.4 Critical appraisal

The risk of bias in included studies was assessed using RoB2 (Cochrane risk-of-bias tool for randomized trials) (8). The overall certainty of the evidence was rated using the Grading of Recommendations, Assessment, Development, and Evaluation (GRADE) approach (9). Initial evaluations were conducted by HSP, and subsequently verified by expert reviewers, MNP and PDC. Any discrepancies were rigorously discussed until a consensus was achieved among the reviewers.

### 2.5 Statistical analysis

We used risk ratios (RR) with 95% confidence intervals (CIs) and random effects models to pooled data. The statistical heterogeneity of meta-analysis estimates was assessed using the I2 statistic. We used STATA V17 for data analysis.

## 3 Results

### 3.1 Study selection

**Figure 1:**
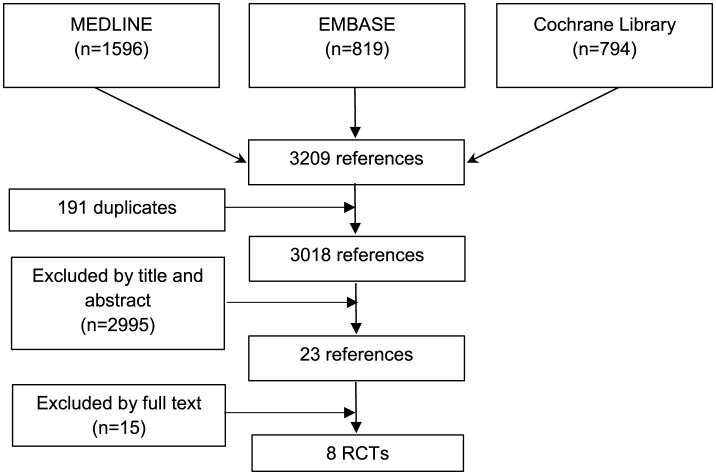
Flowchart of the study selection process.

A total of 3,209 references were identified across several databases–1,596 in Pubmed, 819 in EMBASE, and 794 in the Cochrane Library. After eliminating 191 duplicates, two reviewers initially screened the titles and abstracts of 3,018 references. Through this screening, 2,995 studies were excluded, leaving 23 for full text review (Figure 1). Ultimately, eight RCTs were chosen for inclusion. The references of the excluded studies and the reasons for their exclusion are detailed in Supplementary Material.

### 3.2 Characteristics of the RCTs

The RCTs were published between 2013 and 2023, with most being phase 4 (10–14), except for three that were phase 3 (15–17). Table 1 summarizes the characteristics and risk of bias of the eight studies included.

**Table 1:** Characteristics of the RCT included and risk of bias.

**Study, year**	**Design**	**Inclusion criteria**	**Intervention (*n*)**	**Comparator (*n*)**	**Follow-up**	**Outcome**	**Risk of bias**
Cantini, 2013, (10)	Open label phase 4. Italian. Monocentric	Patients with AS according to NY criteria that are in remission BASDAI <4 in baseline visit at January 2010	Etanercept 50 mg every 2 w (22)	Etanercept 50 mg every week (21)	96 w	% remission (BASDAI <4 and normal acute phase reactants) % flare (BASDAI >4, enthesitis, dactylitis or inflammatory back pain according to Calin criteria)	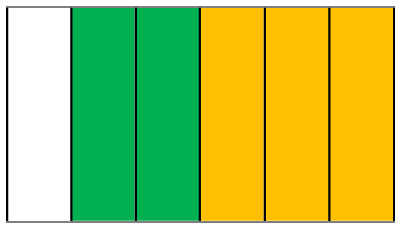
Gratacos, 2019, (11)	Open label phase 4. Spain. Multicentric.	AxSpA patients according to ASAS criteria in remission for at least 6 month previous to inclusion	Adalimumab 40 mg every 3 w, Etanercept 50 mg every 2 w, Golimumab 50 mg every 6 w, Infliximab 3 mg/kg every 6–8 w. (55)	Adalimumab 40 mg every 2 w, Etanercept 50 mg every 1 w, Golimumab 50 mg every 4 w, Infliximab 5 mg/kg every 5–6 w. (58)	48 w	% low disease activity (BASDAI <4, PhGA <4, PGA <4 y VAS <4) % remission (BASDAI ≤ 2, normal CRP and low VAS)	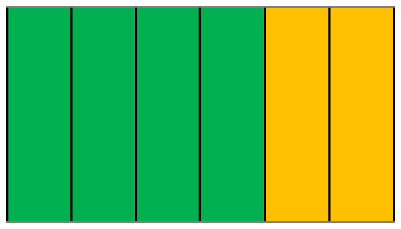
Landewe, 2018, (15)	Double blind	NR AxSpA according to ASAS criteria that has completed Adalimumab treatment during 28 w with ASDAS remission <1, 3 in weeks 16, 20, 24 and 28	Placebo with or without DMARDcs (153)	Adalimumab 40 mg every 2 w with or without DMARDcs. (152)	40 w	% remission without flare (2 or more visit ASDAS ≥2.1) % inactive disease ASDAS <1.3 % flare (2 or more visit ASDAS ≥2.1) % partial flare (2.1 > ASDAS ≥1.3)	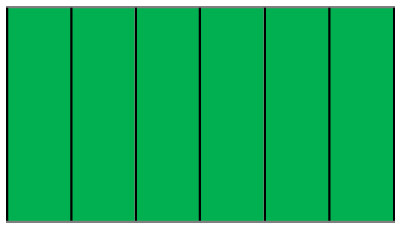
Landewe, 2020, (16)	Double blind	AxSpA according to ASAS or mNY criteria that has completed Certolizumab treatment during 52 w that were in remission with ASDAS <1.3 in at least one visit at week 16 or 20; ASDAS <2.1 in two visits between week 16 and 20	Placebo, 104 patients Certolizumab every 4 w (105)	Certolizumab 200 mg every 2 s, (104)	48 w	% remission without flare (2 or more visit ASDAS ≥2.1 or ASDAS >3.5) % low disease activity (2.1 > ASDAS ≥ 1.3) % inactive disease (ASDAS <1.3) % high disease activity (ASDAS ≥3.5)	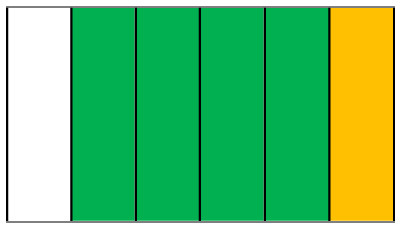
Landewe, 2021, (17)	Double blind	AxSpA according to ASAS or mNY criteria that had completed Ixekizumab treatment during 52 w that were in remission with ASDAS <1.3 in at least one visit at week 16 or 20; ASDAS <2.1 in two visits between week 16 and 20	Placebo, 53 patients. Ixekizumab 80 mg every 4 w (48)	Ixekizumab 80 mg every 2 w (54)	40 w	% remission without flare (2 or more visit ASDAS ≥2.1 or ASDAS > 3.5) % ASDAS worsening ≥0.9 % low disease activity (ASDAS <2.1) % inactive disease (ASDAS <1.3)	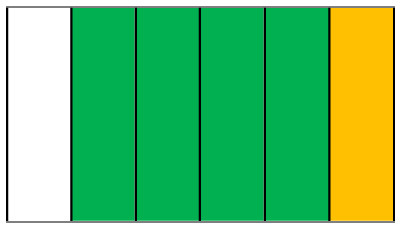
Michielsens, 2022, (14)	Open label. Phase 4. Monocentric. Netherland	AxSpA in low disease activity (ASDAS <2.1 or PASDAS 3.2) during at least 6 months before inclusion	Adalimumab or Certolizumab every 3–4 w, Etanercept every 2–3 w, Golimumab every 6–8 w and Infliximab 1.5–2.25 mg/kg every 8 w until suspension. (39)	Adalimumab 40 mg or Certolizumab 200 mg every 2 w, Etanercept 50 mg every 1 w, Golimumab 50 mg every 4 w, Infliximab 3 mg/kg every 8 w. (19)	48 w	% low disease activity (ASDAS <2.1 without peripheral disease flare)	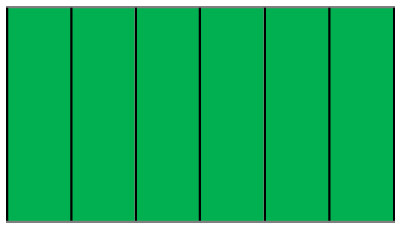
Ruwaard, 2023, (13)	Open label. Phase 4. Monocentric. Netherland	AS patient treated with Etanercept that were in remission with ASDAS <2.1 during at least 6 months before inclusion	Etanercept 50 mg every 2 w with or without DMARDcs. (20)	Etanercept 50 mg every 1 w with or without DMARDcs. (20)	24 w	% low disease activity (ASDAS 2.1)	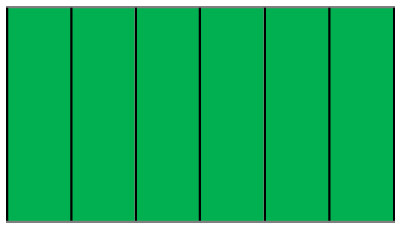
Weinstein 2023 (12)	Double blind. Phase 4. Multicentric	NR AxSpA treated with Golimumab every 4 w during 10 months that with inactive disease ASDAS <1.3 at months 7 and 10 before influsion	Placebo, 62 patients. Golimumab 50 mg every 8 w, (63)	Golimumab 50 mg every 4 w (63)	48 w	% remission without flare (2 or more visit ASDAS ≥2.1 or ASDAS worsening ≥1.1) % low disease activity (ASDAS <2.1) % flare (2 or more visit ASDAS ≥2.1 or ASDAS worsening ≥1.1)	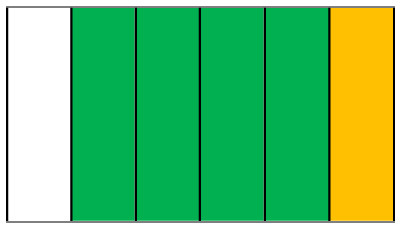

ASAS, Assessment of SpondyloArthritis international Society; AxSpA, Axial Spondyloarthritis; DMARDcs, conventional synthetic DMARDs; mNY, modified New York; W, weeks.

Cochrane Risk of Bias 2 tool: 1 = Bias arising from randomization process; 2 = Bias due to deviations from intended interventions; 3 = Bias due to missing outcome data; 4 = Bias in measurement of outcome; 5 = Bias in selection of reported result; 6 = Overall risk of bias.

In total, 1,286 patients were included across all studies. Participants' ages ranged from 18 to 65 years. The proportion of females varied between 15.9 and 38%. Participants in the studies were primarily selected based on diagnostic criteria from the ASAS or the modified New York criteria, with remission status defined by BASDAI or ASDAS scores at baseline. The specific criteria for remission at inclusion and the precise operational definitions for all primary and secondary outcomes for each study are detailed in Table 2. Two studies included only non-radiographic axial spondyloarthritis (nr-axSpA).

**Table 2:** Remission criteria and flare definition of the studies included.

**Author, Year**	**Definition of remission (or Success)**	**Definition of flare (or Failure)**
Cantini, 2013	Maintaining remission (defined as BASDAI <4 with normal CRP)	Not explicitly defined; reported as the inverse of maintaining remission
Gratacós, 2019	Maintaining ID (BASDAI <2, no active joints, low CRP); Inactive disease (ASDAS <1.3) Low disease activity (BASDAI, VAS, PGA <4)	An absolute ASDAS worsening of ≥1.1
Landewe, 2018	Maintaining remission without a flare Maintaining ID (ASDAS <1.3)	ASDAS ≥2.1 on two consecutive visits A “partial flare” was also defined as 2.1 > ASDAS ≥1.3
Landewe, 2020 Landewe, 2021 Weinstein, 2023	Remaining without a flare Maintaining ID (ASDAS <1.3) Maintaining LDA (ASDAS <2.1)	ASDAS ≥2.1 on two consecutive visits, or an absolute ASDAS worsening of ≥1.1
Michielsen, 2022 Ruwaard, 2022	Maintaining LDA (ASDAS <2.1)	Not explicitly defined as a primary outcome; failure was the inverse of maintaining LDA

CRP, C reactive Protein; ID, inactive disease; HDA, high disease activity; LDA, low disease activity.

Clinically, participants were enrolled in the studies during periods of remission or while exhibiting low disease activity. Disease duration varied depending on inclusion criteria, ranging from newly diagnosed individuals and those with long-standing disease. The interventions involved mostly anti-TNFα treatment except for one trial that involved Ixekizumab.

Detailed description of the demographical and clinical characteristics of the participants is summarized in Table 3.

**Table 3:** Demographic and clinical characteristics of participants.

**Author, Year**	**Population type**	***N***	**Sex, *n* (%)**	**Age**	**Disease duration**	**B27, *n* (%)**	**ASDAS**	**BASDAI**	**CRP**
Cantini, 2013	AS	43	9 (20.9)	NI	13 (NI)^**^	72 (92.3)	NI	6.9 (1.9)	1.9 (1.1)
Gratacos, 2019	AxSpA	120	18 (15.9)	45.6 (13.0)^**^	10.0 (5.9–20.3)^**^	NI	1.1 (0.7; 2.0)^**^	1.0 (0.4; 1.6)^**^	NI
Landewe, 2018,	nr-AxSpA	305	116 (38)	35 (0.2)^*^	1.8 (2.9)^*^	266 (87)	0.7 (0.4)^*^	0.8 (0.7)^*^	1.5 (2.1)^*^
Landewe, 2020	AxSpA <5 years duration	313	222 (30.16)	32.9 (7)^*^	2.2 (1.7)^*^	617 (83.8)	3.7 (0.8)^*^	6.7 (1.4)^*^	NI
Landewe, 2021	AxSpA	155	24 (23.5)	37.5 (10.3)^*^	7.4 (7.7)^*^	92 (90)	1.3 (0.5)^*^	1.5 (1.1)^*^	2.5 (4.3)^*^
Michielsens, 2022	AS	122	21 (36)	NI	NI	52 (89.7)	NI	NI	NI
Ruwaard, 2022	AS	40	7 (17.5)	NI	NI	33 (82.5)	NI	NI	NI
Weinstein, 2023	nr-AxSpA <5 years duration	188	56 (29.8)	31.5 (18–45)^**^	NI	138 (73.4)	3.8 (1.9–6.4)^**^	7.0 (4.0–10.0)^**^	NI

^*^Mean (SD).

^**^Median (IQR).

AS, Ankylosing Spondylitis; AxSpA, axial spondyloarthritis; nr-AxSpA, non-radiographic axial spondyloarthritis; CRP, C reactive Protein.

The follow-up periods ranged from 24 to 96 weeks. The risk of bias was assessed as low in three of the RCTs and remained uncertain in the other five, largely due to incomplete details on randomization processes and allocation concealment.

### 3.3 Efficacy of treatment withdrawal

#### 3.3.1 Patients with sustained remission (at least 6 months)

Only one study, by Landewé et al. (15), assessed the efficacy of treatment withdrawal in patients with at least 6 months prior to inclusion. The study included 305 patients with nr-axSpA and compared the outcomes of withdrawing Adalimumab to continuing with the standard regimen over a period of 10 months.

In this study, it was found that 47.1% patients of the withdrawal group had maintained remission without experiencing a flare, compared to 70.4% patients who continued on the standard regimen (RR 0.66, 95% CI 0.55–0.81; high certainty evidence). Additionally, 33.3% patients of the withdrawal group maintained a state of inactive disease (ASDAS <1.3) as opposed to 57.2% patients on the standard regimen (RR 0.58, 95% CI 0.45–0.76; high certainty evidence).

Conversely, 52.9% patients of the withdrawal group experienced a disease flare (ASDAS ≥2.1 on two consecutive visits) compared to 29.6% patients in the standard regimen group (RR 1.79, 95% CI 1.34–2.38; high certainty evidence). Furthermore, 64.1% patients of the withdrawal group experienced a partial flare (2.1 > ASDAS ≥ 1.3) in contrast to 40.8% patients who continued with the standard regimen (RR 1.57, 95% CI 1.25–1.97; one trial; high certainty evidence).

**Figure 2:**
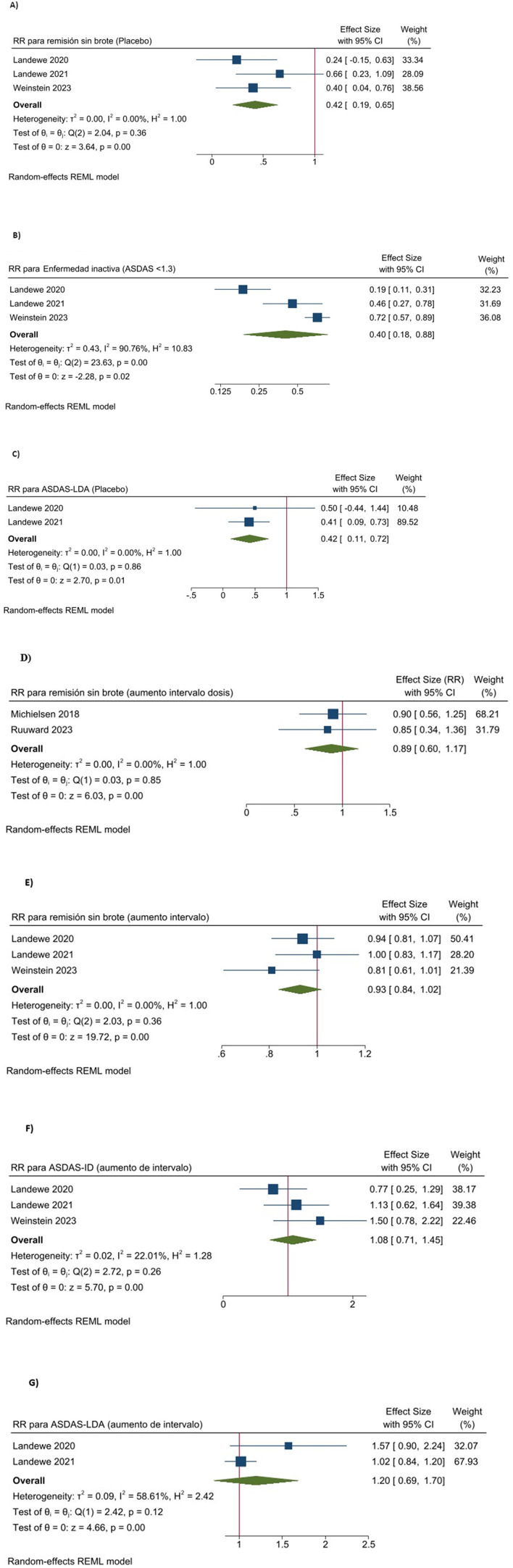
Forest plot of the meta-analysis combining the results of the RCTs. **(A)** Efficacy of treatment discontinuation in patients with short term remission: remission without flare. **(B)** Efficacy of treatment discontinuation in patients with short term remission: inactive disease. **(C)** Efficacy of treatment discontinuation in patients with short term remission: low disease activity. **(D)** Efficacy of treatment tapering in patients with sustained remission: low disease activity. **(E)** Efficacy of treatment tapering in patients with short term remission: remission without flare. **(F)** Efficacy of treatment tapering in patients with short term remission: inactive disease. **(G)** Efficacy of treatment tapering in patients with short term remission: low disease activity.

Table 4 summarizes outcome results and certainty of evidence. Figure 2 shows the forest plot of the meta-analysis of the outcomes.

**Table 4:** Synthesis and certainty of evidence.

**Outcome (*n* studies)**	**Intervention, *n*/*N* (%)**	**Comparator, *n*/*N* (%)**	**RR (95% CI)**	**Risk of bias**	**Inconsistency**	**Indirectness**	**Imprecision**	**Certainty**
**Discontinuation vs standard regimen in patients that were in remission for at least 6 months**								
Remission without flare (1)	72/153 (47.1)	107/152 (70.4)	0.66 (0.55–0.81)	Not serious	Not serious	Not serious	Not serious	⊕⊕⊕⊕ High
Inactive disease (1)	51/153 (33.3)	87/152 (57.2)	0.58 (0.45–0.76)	Not serious	Not serious	Not serious	Not serious	⊕⊕⊕⊕ High
Flare (1)	81/153 (52.9)	45/152 (29.6)	1.79 (1.34–2.38)	Not serious	Not serious	Not serious	Not serious	⊕⊕⊕⊕ High
Partial flare (1)	98/153 (64.1)	62/152 (40.8)	1.57 (1.25–1.97)	Not serious	Not serious	Not serious	Not serious	⊕⊕⊕⊕ High
**Discontinuation vs standard regimen in patients that were in remission for**<**6 months**								
Remission without flare (3)	71/219 (32.4)	185/221 (83.7)	RR 0.41 (0.19–0.65)	Serious	Not serious	Not serious	Not serious	⊕⊕⊕○ Moderate
Remission without worsening in ASDAS (1)	16/53 (30.2)	40/54 (74.1)	0.41 (0.26–0.63)	Serious	Not serious	Not serious	Serious	⊕⊕○○ Low
Inactive disease (3)	65/219 (29.7)	158/221 (71.5)	0.4 (0.18–0.88)	Serious	Not serious	Not serious	Not serious	⊕⊕⊕○ Moderate
Low disease activity (2)	44/157 (28)	131/158 (82.9)	0.36 (0.15–0.86)	Serious	Not serious	Not serious	Not serious	⊕⊕⊕○ Moderate
Flare (1)	38/62 (61.3)	10/62 (16.1)	3.86 (2.12–7.05)	Serious	Not serious	Not serious	Serious	⊕⊕○○ Low
**Tapering vs standard regimen in patients that were in remission for at least 6 months**								
Inactive disease BASDAI (1)	78.2^*^	83.7^*^	−5.5 (−20.6 to 9.7)^*^†	Serious	Not serious	Not serious	Serious	⊕⊕○○ Low
Inactive disease ASDAS (1)	53.5^*^	61.4^*^	7.8 (−10 to 25.8)^*^†	Serious	Not serious	Not serious	Serious	⊕⊕○○ Low
Low disease activity BASDAI (1)	81.3^*^	83.8^*^	−2.5 (−16.6 to 11.7)^*^†	Serious	Not serious	Not serious	Serious	⊕⊕○○ Low
Low disease activity by ASDAS (2)	37/59 (62.7)	32/39 (82.1)	0.89 (0.66–1.18)	Not serious	Not serious	Not serious	Serious	⊕⊕⊕○ Moderate
Flare ASDAS (1)	12.7^*^	6.6^*^	−6.1 (−25.2 to 12.9)^*^†	Serious	Not serious	Not serious	Serious	⊕⊕○○ Low
**Tapering vs standard regimen in patients that were in remission for**<**6 months**								
Remission without flare (3)	166/216 (76.9)	185/221 (38.7)	0.93 (0.84–1.02)	Serious	Not serious	Not serious	Not serious	⊕⊕⊕○ Moderate
Remission without ASDAS worsening (1)	35/48 (72.9)	40/54 (74.1)	0.98 (0.78–1.24)	Serious	Not serious	Not serious	Serious	⊕⊕○○ Low
Inactive disease (3)	140/216 (64.8)	158/221 (71.5)	1.08 (0.71–1.45)	Serious	Not serious	Not serious	Not serious	⊕⊕⊕○ Moderate
Low disease activity (2)	117/153 (76.5)	131/158 (82.9)	0.94 (0.81–1.09)	Serious	Not serious	Not serious	Not serious	⊕⊕⊕○ Moderate
Flare (1)	15/63 (23.8)	10/63 (15.9)	1.5 (0.73–3.08)	Serious	Not serious	Not serious	Serious	⊕⊕○○ Low
**Tapering vs standard regime in patients in remission at inclusion (duration not specified)**								
Remission (1)	19/22 (86.4)	19/21 (90.5)	0.95 (0.77–1.18)	Serious	Not serious	Not serious	Serious	⊕⊕○○ Low
Flare (1)	3/22 (13.6)	2/21 (9.5)	1.43 (0.27–7.73)	Serious	Not serious	Not serious	Serious	⊕⊕○○ Low

^*^Expressed as percentages. Absolute values (n/N) and RR not available from the data provided by the publication.

^†^The publication provided the risk difference instead of the RR.

#### 3.3.2 Patients with short term remission (<6 months)

Three double-blind RCTs—Landewé et al. (16, 17), and Weinstein et al. (12)—assessed the efficacy of treatment withdrawal in maintaining remission in 440 patients with axSpA who had achieved sustained remission between months 4 and 10 post-treatment initiation. These studies evaluated the withdrawal of Certolizumab (16), Ixekizumab (17), and Golimumab (12) compared to the standard regimen over a follow-up period of 10–12 months.

Pooled analysis of the three studies (12, 16, 17) showed that 32.4% patients of the withdrawal group remained without a flare (defined as ≥ASDAS 2.1 on two consecutive visits or an ASDAS worsening ≥1.1), compared to 83.7% patients of the standard regimen group (RR 0.41, 95% CI 0.19–0.65; three trials; moderate certainty evidence; Table 4, Figure 2A).

Landewé et al. (17), observed that 30.2% patients of the withdrawal group remained in remission without an ASDAS worsening ≥0.9 compared to 74.1% patients in the standard regimen group (RR 0.41, 95% CI 0.26–0.63; one trial; low certainty evidence).

Moreover, according to the three RCTs (12, 16, 17), 29.7% patients of the withdrawal group remained in an inactive disease state (ASDAS <1.3) compared to 71.5% patients of the standard regimen group (RR 0.40, 95% CI 0.18–0.88; three trials; moderate certainty evidence; Table 4, Figure 2B).

Additionally, according to Landewe et al. (16, 17), 28% patients of the withdrawal group maintained low disease activity (ASDAS <2.1) as opposed to 82.9% patients on standard regimen group (RR 0.36, 95% CI 0.15–0.86; two trials; moderate certainty evidence; Table 4, Figure 2C).

In the study of Weinstein et al. (12), 61.3% (38/62) patients of the withdrawal group experienced a flare (≥2 consecutive ASDAS ≥2.1 or worsening ≥1.1) compared to 16.1% patients of the standard regimen group (RR 3.86, 95% CI 2.12–7.05; one trial; low certainty evidence).

### 3.4 Efficacy of treatment tapering

#### 3.4.1 Patients with sustained remission (at least 6 months)

Three open randomized clinical trials—Gratacós et al. (11), Michielsens et al. (14), and Ruwaard et al. (13)—explored the efficacy of treatment tapering in maintaining remission among 211 patients with axSpA who had been in sustained remission for at least 6 months. The studies focused on tapering anti-TNFα treatment (11, 14) and Etanercept (13) compared to the standard regimen over a follow-up period of 6–12 months.

According Gratacos et al. (11), 78.2% of patients in the treatment tapering group maintained inactive disease status according to BASDAI (characterized by BASDAI <2, no joint activity, and low CRP) compared to 83.7% in the standard regimen group, with no significant difference (risk difference −5.5%, 95% CI −20.6% to 9.7%; 1 trial; low certainty evidence). Furthermore, 53.5% of the tapering group maintained inactive disease status according to ASAS (ASDAS <1.3) vs. 61.4% in the standard regimen group, with no significant difference (risk difference 7.8%, 95% CI −10% to 25.8%; 1 trial; low certainty evidence) (11). The same study revealed that 81.3% patients remained with low disease activity (defined by BASDAI, VAS, and PGA <4) after tapering, vs. 83.8% patients in the standard regimen group, with no significant difference (risk difference −2.5%, 95% CI −16.6% to 11.7%; one trial; low certainty evidence) (11). Regarding flares, Gratacos et al. (11) observed that 12.7% of the tapering group experienced a new flare (ASDAS worsening ≥1.1) compared to 6.6% in the standard regimen group, with no significant difference (risk difference −6.1%, 95% CI −25.2% to 12.9%; low certainty evidence).

According to Michielsens et al. and Ruwaard et al. (13, 14), 62.7% of the patients in the tapering groups maintained low disease activity by ASDAS compared to 82.1% in the standard regimen groups, with a non-significant lower risk in the tapering groups (RR 0.89, 95% CI 0.66–1.18; two trials; moderate certainty evidence; Table 4, Figure 2D).

#### 3.4.2 Patients with short-term remission (<6 months)

Three double-blind RCTs—Landewe et al. (16, 17), and Weinstein et al. (12)—investigated the efficacy of treatment tapering in maintaining remission in 437 patients with axSpA who had achieved sustained remission between months 4 and 10 post-treatment initiation. These studies compared tapering Certolizumab, Ixekizumab, and Golimumab against a standard regimen over a follow-up period of 10–12 months.

Pooled analysis of the three RCTs (12, 16, 17), showed that 76.9% of patients in the treatment tapering groups remained in remission without a flare, compared to 83.7% patients with the standard regimen, suggesting slightly lower but non-significant risk for tapering (RR 0.93, 95% CI 0.84–1.02; three trials; moderate certainty evidence; Table 4, Figure 2E).

It was observed by Landewe et al. (17) that 72.9% patients of the tapering group remained in remission without ASDAS worsening compared to 74.1% patients with the standard regimen, indicating a marginally higher but non-significant risk for tapering (RR 0.98, 95% CI 0.78–1.24; one trial; low certainty evidence).

Regarding inactive disease status, all three studies (12, 16, 17), showed that 64.8% of the patients in the tapering groups kept an ASDAS <1.3, compared to 71.5% patients with the standard regimen, indicating a slightly higher but non-significant risk for tapering (RR 1.08, 95% CI 0.71–1.45; three trials; moderate certainty evidence; Table 4, Figure 2F).

Regarding low disease activity, Landewe et al. (16, 17), showed that 76.5% of patients in the tapering groups maintained an ASDAS <2.1, compared to 82.9% with the standard regimen, with a slight but non-significant decrease in risk associated with tapering (RR 0.94, 95% CI 0.81–1.09; two trials; moderate certainty evidence; Table 4, Figure 2G).

Regarding flares, only Weinstein et al. found that, 23.8% of patients in the tapering group experienced a flare compared to 15.9% with the standard regimen, suggesting a higher but non-significant risk with tapering (RR 1.5, 95% CI 0.73–3.08; one trial; low certainty evidence) (12).

#### 3.4.3 Patients in remission at inclusion (duration not specified)

Only one trial by Cantini et al. (10) assessed the efficacy of treatment tapering in 43 patients with axSpA who were in remission (defined as BASDAI <4 with normal CRP) at the time of inclusion, though the duration of remission prior to the study was not specified. This study compared the outcomes of Etanercept tapering against a standard regimen over a follow-up period of 24 months.

In this study 86.3% of patients in the tapering group had maintained remission compared to 90.4% in the standard regimen group, suggesting a non-significant minor risk for tapering (RR 0.95, 95% CI 0.77–1.18; one trial; low certainty evidence) (10). Regarding flares, 13.6% of patients in the tapering group experienced a flare compared to 9.5% in the standard regimen group, with a non-significant higher risk for tapering (RR 1.43, 95% CI 0.27–7.73; one trial; low certainty evidence).

## 4 Discussion

The current meta-analysis aimed to determine whether withdrawal or tapering of bDMARD in comparison to the standard regimen, could maintain remission or low disease activity in axSpA patients who had already achieved these states. The results showed that treatment withdrawal significantly decreased the likelihood of maintaining remission with a RR of 0.66 (95% CI 0.55–0.81). In contrast, tapering treatment only slightly reduced the probability of sustaining remission with an RR of 0.93 (95% CI 0.84–1.02), which was not statistically significant.

In terms of flare risk, withdrawal was associated with a significantly higher risk RR 1.79 (95% CI 1.34–2.38), while tapering resulted in non-significant increase in flare risk RR 1.5 (95% CI 0.79–3.08). These outcomes were consistent across all subgroups, regardless of whether patients had been in remission for more than 6 months, <6 months, or were in remission at the time of inclusion. Notably, patients with shorter remission durations at inclusion showed an increased flare risk upon withdrawal. However, flare risk remained stable across all subgroups during tapering, irrespective of the remission duration at inclusion. The current meta-analysis extends previous research on the withdrawal or tapering of biological DMARDs in rheumatic diseases. Our findings are consistent with several other analyses that support de-escalation. A SLR published in 2019 by Navarro-Compán et al. (3), which primarily synthesized observational studies and included only one RCT with biologic therapy, also found that tapering strategies may maintain remission or low disease activity.

Webers et al. (4) performed a SLR that informed the 2022 ASAS-EULAR recommendations for axSpA, focusing on flare risks associated with bDMARD withdrawal. The findings from this study found that withdrawal leads to higher flare risks. Tapering was not evaluated. Qualitative studies have shown a broad range of attitudes toward tapering among both patients and physicians (18, 19). The present study contributes to evidence-based decision-making for both physicians and patients, which is important given the lack of biomarkers of remission or flare. Similar results was also observed more recently by Balay-Dustrude et al. (20), the latter performing also a qualitative synthesis of two RCTs and six observational studies.

In contrast, other meta-analyses have reported different results, though methodological differences in study populations and interventions likely explain these discrepancies. An earlier meta-analysis by Uhrenholdt et al. (5), which included randomized controlled trials (RCTs) up to 2019 with participants having rheumatoid arthritis (RA) or spondyloarthritis (SpA), revealed that the risk of flare was increased for both tapering and withdrawing treatment compared to a standard regimen, with relative risks (RR) of 1.45 and 2.28, respectively. A sensitivity analysis showed that the risk of flare was higher in patients with RA than in those with SpA. It is possible that the differences observed compared to our study may be due to patients with SpA being more likely to maintain remission than those with RA, a finding that is also supported by more recent studies published after 2019.

Additionally, a meta-analysis of RCTs conducted by Lawson et al. (21) included some studies that were excluded from our analysis due to differences in inclusion criteria, such as population with active disease state or differing outcome measures. This study concluded that dose reduction had higher risk of relapse and disease flare.

Finally, the meta-analysis by Min et al. (22) concluded that dose reduction could increase flare risk, but their analysis combined data from distinct withdrawal and dose-reduction strategies across various SpA subtypes, which may have led to conclusions that are difficult to interpret when assessing tapering alone.

A significant finding of our analysis is the robustness of the outcomes, which remained consistent despite heterogeneity in both patient populations and disease activity definitions used across the source trials. Our review included trials enrolling patients across the axSpA spectrum, including radiographic, non-radiographic, and mixed cohorts. Although a formal subgroup analysis was precluded by heterogeneity, the data of the individual studies revealed no clear differences in de-escalation success, a finding that supports the current view of axSpA as a single disease entity (23). Similarly, outcomes were comparable whether remission was defined by various ASDAS thresholds or by the established BASDAI <4 standard. That our central findings—the significant risk of flare upon withdrawal and the relative safety of tapering—held true across these varied methodologies suggests the observed effect is a genuine clinical phenomenon, not an artifact of a specific patient subgroup or measurement tool.

Further strengths of our meta-analysis include the deliberate decision to include only randomized controlled trials (RCTs). By focusing exclusively on this highest level of evidence, we aimed to minimize the potential for selection bias inherent in observational data. Furthermore, by segregating the analysis based on previous remission duration, we took a clinical approach that other meta-analyses may have missed due to limited available data. Across most domains, our analysis demonstrated moderate to high certainty, suggesting our conclusions are fairly reliable and robust.

Despite these strengths, some limitations should be considered. Some of the included studies exhibited an unclear risk of bias, primarily stemming from inadequate information regarding the randomization sequence. Furthermore, certain outcomes were associated with low certainty, primarily due to limited sample sizes in some studies; to mitigate this limitation, efforts were made for rigorous analysis and cautious interpretation of the findings.

This established consistency provides a solid foundation for future research, which can now address more nuanced questions. Future studies should investigate the most efficacious treatment tapering strategy, identify prognostic factors for disease remission, and focus on populations with longer sustained remission to enhance understanding and improve management of long-term outcomes. With the emergence of refined assessment tools, such as the new data-driven BASDAI cut-offs proposed by Georgiadis et al. (24), the field is also positioned to investigate whether the depth of remission can predict de-escalation success. Finally, several highly anticipated trials—including SPACING (NCT01610947), SPARTACUS (NCT04435288), BIOTAPE (NCT05115903), and TAPER (NCT04429776)—are expected to provide definitive data on which patients are the optimal candidates for these refined de-escalation strategies. In conclusion, this meta-analysis demonstrates that withdrawal significantly compromises the ability to sustain remission, whereas tapering appears to be a potentially safer alternative, regardless of the duration of prior remission.

## Data Availability

The raw data supporting the conclusions of this article will be made available by the authors, without undue reservation.
